# Age-Related Differences in Intermuscular Coherence EMG-EMG of Ankle Joint Antagonist Muscle Activity during Maximal Leaning

**DOI:** 10.3390/s22197527

**Published:** 2022-10-04

**Authors:** Mariusz Konieczny, Przemysław Domaszewski, Elżbieta Skorupska, Zbigniew Borysiuk, Kajetan J. Słomka

**Affiliations:** 1 Faculty of Physical Education and Physiotherapy, Opole University of Technology, 45-068 Opole, Poland; 2 Department of Health Sciences, Institute of Health Sciences, University of Opole, 45-060 Opole, Poland; 3 Department of Physiotherapy, Poznan University of Medical Sciences, 61-701 Poznan, Poland; 4 Institute of Sport Sciences, Academy of Physical Education, 40-065 Katowice, Poland

**Keywords:** intermuscular coherence, surface electromyography, limit of stability, aging

## Abstract

Background: Intermuscular synchronization is one of the fundamental aspects of maintaining a stable posture and is of great importance in the aging process. This study aimed to assess muscle synchronization and postural stabilizer asymmetry during quiet standing and the limits of stability using wavelet analysis. Intermuscular synchrony and antagonistic sEMG-sEMG (surface electromyography) coherence asymmetry were evaluated in the tibialis anterior and soleus muscles. Methods: The study involved 20 elderly (aged 65 ± 3.6) and 20 young (aged 21 ± 1.3) subjects. The task was to perform a maximum forward bend in a standing position. The prone test was divided into three phases: quiet standing (10 s), dynamic learning, and maintenance of maximum leaning (20 s). Wavelet analysis of coherence was performed in the delta and beta bands. Results: Young subjects modulated interface coherences to a greater extent in the beta band. Analysis of postural stability during standing tasks showed that only the parameter R2b (the distance between the maximal and minimal position central of pressure), as an indicator for assessing the practical limits of stability, was found to be significantly associated with differences in aging. Conclusion: The results showed differences in the beta and delta band oscillations between young and older subjects in a postural task involving standing quietly and leaning forward.

## 1. Introduction

During the aging period, there is an increasing incidence of postural-related disorders, which in the literature is referred to as so-called physiological atrophy of motor performance [1]. This is a phenomenon probably caused not only by changes in peripheral structures, but also by changes in the central nervous system. Understanding the neural control of standing balance is important to identify age-related degeneration. The standing posture of the human body can be biomechanically described as an inverted pendulum rotating about the ankle joint. To prevent falls, an adequate level of ankle stiffness is required, which is provided by the muscle-tendon unit. However, the neuromuscular system must significantly assist this process by actively modulating it to an appropriate level [2]. Successful integration of vestibular and somatosensory systems is required for optimal balance control, but age negatively affects the functioning of these systems [3]. An analysis that may help to explain this phenomenon is a simple calculation of the coherence of a pair of antagonistic muscles (tibialis anterior muscle and soleus muscle), which are relevant in investigating neuromuscular mechanisms controlling the ankle joint. The relationships that exist between the muscles mentioned above have already been analyzed by a number of authors [2,3,4]. However, due to the complexity of the ankle joint stability problem, further exploration of this topic is still needed, if only due to the increase in life expectancy. While many research papers have so far concentrated on peripheral mechanisms, in recent years, research has tended to focus on central mechanisms. Studies related to the thickness of the primary motor cortex indicate a linear correlation between human age and physiological atrophy of these brain areas [5,6,7]. However, the research related to brain atrophy in aging itself does not clearly show significant changes related to their effects on muscle bioelectrical activity and associated impairments contributing to coordination deficits in older people [8,9] and the proprioceptive system [10]. In the process of aging, there is also a risk associated with falls, which are a serious health problem and one of the main causes of injury and disability. It is estimated that one-third of community-dwelling older people experience at least one fall per year [11,12], and research conducted in this area shows that more than half of the causes of falls are related to factors concerning postural control [13]. Postural control, which is understood as a complex relationship existing between the sensory and motor systems [14,15], concerns, among other things, the appropriate perception of environmental stimuli, the response to changes in body orientation in space, as well as the correct maintenance of the central of pressure (COP). Older people have deficits in coordination of movement, resulting in slower and less fluid movements and affecting frequent loss of balance. Difficulties associated with balance are associated with poorer control of COP displacement [15]. The current level of technology allows a number of tools to be used to study postural stability and muscle bioelectrical activity with simultaneous EEG (electroencephalography) analysis; however, the interpretation of the results is not yet completely clear. One method of analysis combining the above aspects is coherence, regarded as the correlation measure between the frequency domain representations of different signals [16]. Other authors describe coherence as an indicator of the linear connection between two signals [17] and as an extension of the Pearson correlation coefficient in the frequency domain [18]. Coherence analysis is now considered as a method for assessing neuromuscular systems and obtaining information about the communication between central and peripheral systems in the control of motor activity [19]. Coherence analysis determined at different frequencies can provide information on the function of the nervous system in controlling muscle activity during the performance of various tasks [20,21], which is particularly related to the development of disorders in aging. The nature of brain waves depends on the activity being performed, and the range of wave frequencies changes many times throughout the day, along with our activity. The authors of the present study focused on the delta and beta wave bands. There is no definitive evidence for the origin of 0.5–5 Hz delta band activity in the brain, but several studies place the site of delta band generation in the cortical areas and also highlight the importance of the coherence EMG-EMG of the delta bands during postural tasks and during decision-making processes [22,23,24]. Studies show that delta band levels decrease with age, which may be related to the aging process [25]. Moreover, delta band consistency may be related to the variability of the force generated by the muscles and thus to postural sway [26]. Another frequency band of muscle activity investigated is the beta band (15–35 Hz). Studies have shown that submaximal voluntary isometric contractions are characterized by a dominant synchrony at around 15–35 Hz [27,28]. Beta band oscillations are clearly observed in EEG recordings from the cerebral motor cortex [29]. Previous scientific studies indicate significant beta band coherence between sensorimotor cortex and muscle contraction in both primates and human beings [3]. Other authors state that slow EEG oscillations delta band can contain neural information regarding the postural kinematics of the human body [30]. The importance of asymmetries in terms of intermuscular coherence is also a phenomenon that has been little studied in terms of balance. Few scientific papers have been written describing the location of oscillations of different bands and their influence on balance tasks [30,31,32,33]. In the context of the presented research, we hypothesize that, due to aging processes, there are differences in the oscillation of coherences in terms of beta and delta wave frequencies, due to the type of postural stability task performed. Furthermore, the second hypothesis is that there is a reduction in LoS (limits of stability) with age, determined by the R2b index (the distance between the maximal and minimal position of central of pressure anterior/posterior in the 2nd phase). Therefore, the aim of this study is to determine the level of coherence asymmetry in the delta and beta bands during limits of stability in association with aging. This has important implications for determining the changes associated with the phenomenon known as physiological atrophy.

## 2. Materials and Methods

A total of 44 people in two groups participated in the study. The senior group included 22 people aged 65 ± 3.6 (members of the senior citizens’ association). Participation was preceded by prior recruitment carried out in the community of Opole senior citizens. The young people’s group consisted of 22 healthy students aged 21 ± 1.3. 

### 2.1. Applied Equipment 

Muscle bioelectrical activity measurement (sEMG) was performed using a TeleMyo DTS surface electromyography (Noraxon), following the SENIAM methodology (Surface ElectroMyoGraphy for the Non-Invasive Assessment of Muscles). We recorded electromyographic (sEMG) activity from the tibialis anterior muscle (TA) and soleus muscle (SOL) of both lower limbs. Before the study, the electrode sticking site was prepared by removing hair and cleaning the skin to improve electrode adhesion. Surface electrodes (Ag/AgCl) were placed on the muscle belly between the motor point and the tendon attachment, along the longitudinal midline of the muscle. The TeleMyo DTS system (Noraxon) had the following technical specifications: device fundamental noise of less than 1 μV RMS, input impedance greater than 100 MΩ, CMR (common-mode rejection ratio) greater than 100 dB, sampling frequency 1500 Hz, and gain 500. Matlab software was used to analyze the signals [34].

The registration of the standard deviation (SD) of central of pressure (COP) displacements was performed by a force plate (type: 9286AA; Kistler Group, Winterthur, Switzerland), with a sampling frequency of 100 Hz and the duration of the test equal to 30 s [35]

### 2.2. Inclusion Criteria

The following inclusion criteria were adopted during the study: ability to comprehend commands (based on mini mental state examination <23 points) and lack of lower limb injury and medical contraindications to participate in moderate physical exercise, and an agreement to take over the role of test subject.

#### Exclusion Criteria

The criteria adopted as the basis for the potential rejection of an application included: aphasia, significant loss of sight or hearing, which makes it impossible to assess cognitive functions, as well as voluntary resignation from participation in the study. The participants of the study signed the written informed consent. The goal of the study was approved by the Bioethics Committee of the Chamber of Physicians (Resolution No. 237 of 13 December 2016) in accordance with the guidelines described in the Declaration of Helsinki involving human beings [35]. 

### 2.3. Study Protocol

The task consisted of a leaning test preceded by quiet standing. The test performed was divided into three phases: (i) quiet standing, (ii) dynamic leaning, (iii) maintenance of maximum leaning. The start and end of the leaning—the phase boundaries—were determined using the Matlab function “findchangepts” with a linear trend change parameter in the signal. The function uses as the total deviation the sum of the squared differences between the signal values and the predictions of the least-squares linear fit through the values. 

### 2.4. Central of Pressure and Coherence Analysis

The SD of COP displacements was calculated for the quiet standing task and for the maximum forward leaning (limits of stability—LOS) task. For the first and third phases, the SD of COP displacements were calculated (for the first and last 9 s of each phase). Additionally, the R2b (the distance between the maximal and minimal position of central of pressure anterior/posterior in the 2nd phase) parameter was estimated in order to assess the effective stability limits between the mean values of COPA/P trajectory in the 1st and 3rd phases of the trial [36].

We quantified the following: (1) COP variability as the standard deviation (SD) of anterior-posterior COP displacements (in the AP planes); (2) COP modulation as the power of COP displacements from 0 to 2 Hz. [27] Coherence analysis was carried out in two frequency bands 0–5 Hz and 15–35 Hz, (in the first and third phases). Mean coherence values were calculated in the same way as for COP, i.e., over the time interval of the first and last 9 s of each phase. Coherence was determined between the TA-SOL muscle pair separately for both limbs in the study groups and was calculated using the Matlab version R2021a function “wcoherence”. 

The wavelet coherence of two time series *x* and *y* is:

$$\frac{|S\left(C_{x}^{*}\left(a,b\right)C_{y}\left(a,b\right)\right){|}^{2}}{S(|C_{x}\left(a,b\right){|}^{2})\cdot S(|C_{y}\left(a,b\right){|}^{2})}$$

*C_x_*(*a*,*b*) and *C_y_*(*a*,*b*) denote the continuous wavelet transforms of *x* and *y* at scales *a* and positions *b*. The superscript * is the complex conjugate and *S* is a smoothing operator in time and scale.

For real-valued time series, the wavelet coherence is real-valued if you use a real-valued analyzing wavelet, and complex-valued if you use a complex-valued analyzing wavelet [37,38]. 

### 2.5. Statistical Analysis

The collected data were analyzed by application of Jamovi 1.1.9. software. In this study, the authors used ANOVA to analyze the significance of the differences between groups. The Independent Samples *T*-Test was used to determine the intergroup differences in the R2b parameter. The sample size of 22 (in each group) participants in 2 groups is sensitive enough to detect effect size f = 0.4 (η²_p_ = 0,16) power 80% and a 5% (two-sided) significance level. Due to the skewed distributions of each parameter, a logarithmic (natural base) transformation was applied.

## 3. Results

**Figure 1:**
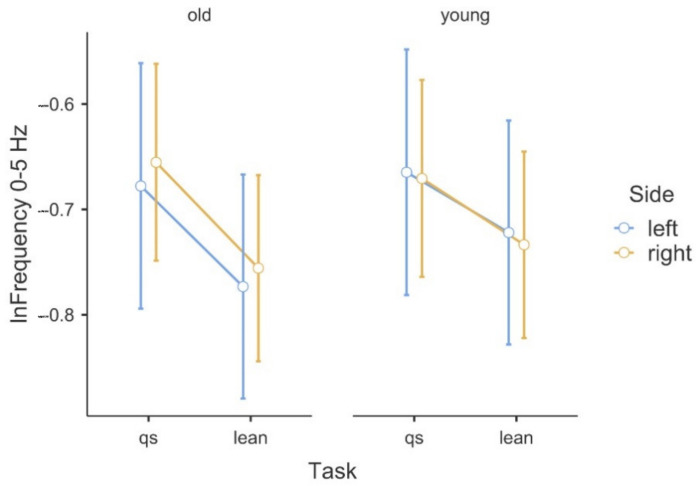
Illustration of the asymmetry of the logarithmic delta band frequency data in the (qs/lean) tasks in the comparison of the two groups.

**Figure 2:**
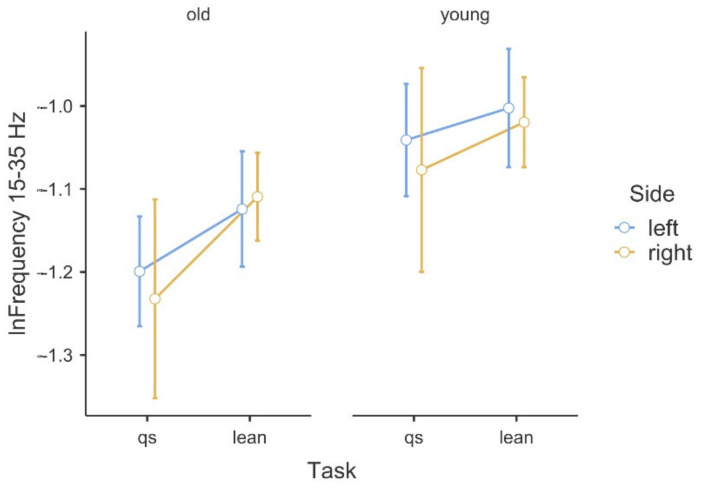
Illustration of the asymmetry of the logarithmic beta band frequency data in the (qs/lean) tasks in the comparison of the two groups.

**Figure 3:**
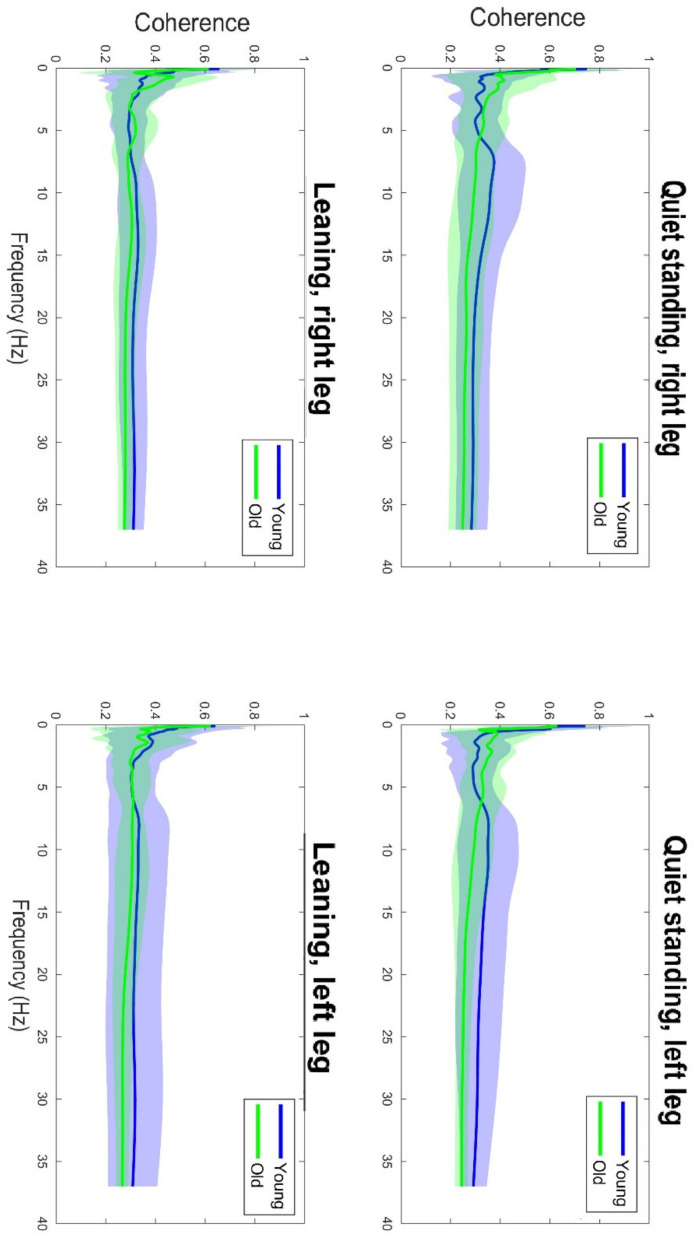
Graphical illustration of the asymmetry (left/right leg) of the delta and beta band coherence oscillations in the tasks (qs/leaning) in both groups.

Table 1 shows the results of the ANOVA analysis of the significance of the Within Subjects Effects differences in the delta band (0–5 Hz) and beta band (15–35 Hz). Within the delta band, statistically significant differences at F(1,42) = 6.78, *p* ≤ 0.01 occurred only between the Within Subjects Effects TASK (qs/lean). The statistical significance presented demonstrates the fact that there is significant variability in the frequency of the delta band waves, due to the type of task, regardless of age group. Coherence in the delta band is also not modulated in terms of body asymmetry, and there is no interaction between Task x Side x GROUP. A graphical analysis of the differences is shown in Figure 1. A statistical analysis of the beta band (15–35 Hz) shows significant differences between both the Within Subjects Effects TASK (qs/lean) at the level of F(1,42) = 10.41, *p* ≤ 0.001 as well as significant differences F(1,42) = 10.60, *p* ≤ 0.001 between the Between Subjects Effects GROUP (young/old). In the analyzed band, the effect of young age on the modulation of coherence is evident, which is associated with an increase in coherence values. The values shown also demonstrate the fact that the frequency of this band increases during the body leaning task regardless of age. Such significant modulation of the delta band frequencies may also be indicative of age-related degenerative changes, which may influence occurrences with decreased body control. A graphical analysis of the differences is presented in Figure 2. The authors found no statistically significant difference in coherence asymmetry in both analyzed bands between young and older participants. The results of the significance analysis of the differences of the R2b parameter, between the study group of young and elderly people, are presented in Table 2, while the SD parameter is presented in Table 3. The results of the descriptive statistics of the parameters studied are shown in Table 4. A graphical analysis of the coherence oscillations in the asymmetry of the two groups is shown in Figure 3.

## 4. Discussion

In the present study, wavelet analysis was used to determine intermuscular coherence in order to identify differences in intermuscular synchronization and lower limb muscle asymmetry in relation to aging processes. Our analysis of intermuscular coherence in the delta and beta bands (tibialis anterior and soleus muscles) in terms of asymmetry in the quiet standing and leaning task was related to the R2b factor and the SD of COP displacements to show the difference between young and older people.

Our analysis of intermuscular coherence in the delta and beta bands (tibialis anterior muscle and soleus muscle) in terms of asymmetry in the silent standing and leaning task was related to the SD of COP displacements and was designed to show the difference between young and elderly people. The R2b parameter was estimated in order to assess the effective stability limits between the mean values of the COPA/P trajectory between qs/lean. We present that the differences in intermuscular coherence that occur during the aging process and its impact on imbalance are also confirmed by studies by other authors [24,39], and that they are linked to changes in the nervous system. In the scientific reports cited in this article, the authors conclude that there is a progressive and heterogeneous deterioration in the functioning of the sensorimotor and neuromuscular systems with age. They conclude by hypothesizing that aging is associated with a reorganization of postural muscle control mechanisms. In our study, there was no statistic asymmetry phenomenon; however, in terms of the delta band oscillations, increased differences between the logarithmic values of the right and left limb in the elderly were noticeable. The delta band oscillation changes markedly due to the difficulty of the task without significant differences related to asymmetry and age. [30] also recognizes the importance of increased cortical activation in the delta band during difficult postural conditions. However, the studies related to statistically significant age-related delta band differences that it presents [39] are not confirmed. It is also important to note that many studies vary by the type of muscles analyzed and the tasks performed. Clark [40] finds an association of changes in the delta band oscillations, but this relates to the synergistic muscles during gait. There are reports in the scientific literature that differentiate age-related asymmetry in the lower limbs in association with a decrease in lower limb muscle strength, without being able to pinpoint a cause [24,41,42,43]. In the scientific reports cited in this article, the authors conclude that there is a progressive and heterogeneous deterioration in the functioning of the sensorimotor and neuromuscular systems with age. They conclude the paper by hypothesizing that aging is associated with a reorganization of the synergistic mechanisms controlling postural muscles. The result of a recent study [44] confirms that, under the influence of training associated with EMG biofeedback, one can voluntarily increase intermuscular coherence, and thus synchronization of the vastus muscles. 

The results of our study on the relationship between postural stability and aging show that the R2b index changes significantly with aging, as younger people are able to lean further forward. This is corroborated by the results of other authors, who find a significantly smaller range of limits of stability in older people, possibly related to the phenomenon of natural belay and fear of falls [13,45,46,47]. Tomita [13] also confirms in his study that older people show a reduction in the range of stability in a number of passages, which also supports our hypothesis of a reduction in the stability limit of older people.

## 5. Conclusions

In the present study, we demonstrated differences in the beta and delta band oscillations between young and older subjects in a postural task involving quiet standing and forward leaning. Young subjects modulated intermuscle coherences to a greater extent in the beta band, which may indicate the presence of age-related detection of changes in multi-muscular control, but there were no differences in intermuscular asymmetry. Our study demonstrates the importance and potential of using intermuscular coherence analysis as an indicator of synchronization between cortical motor regions and their associated body muscles. Analysis of postural stability during standing tasks showed that only the parameter R2b, as an indicator for assessing the effective limits of stability, was found to be significantly associated with differences in aging.

## Figures and Tables

**Table 1:** Results of ANOVA analysis of significance of Within Subjects Effects differences in delta band (0–5 Hz) and beta band (15–35 Hz).

	F	*p*	η²_p_
delta band (0–5 Hz)			
Task	6.78	0.013	0.14
Task × GROUP	0.39	0.536	0.01
Side	0.03	0.867	0.00
Side × GROUP	0.19	0.669	0.00
Task × Side	0.01	0.919	0.00
Task × Side × GROUP	0.00	0.994	0.00
GROUP	0.13	0.717	0.00
beta band (15–35 Hz)			
Task	10.41	0.003	0.04
Task × GROUP	1.25	0.270	0.00
Side	0.59	0.446	0.00
Side × GROUP	0.14	0.708	0.00
Task × Side	0.45	0.509	0.00
Task × Side × GROUP	0.08	0.774	0.00
GROUP	10.60	0.002	0.21

F—*F*-test value (ANOVA); *p*—*p*-value—level of statistical significance (set at 0.05); η²_p_—partial eta squared—determines the effect size of variables in ANOVA models.

**Table 2:** Results of the significance analysis of differences of the R2b parameter, between the study group of young and older people, analyzed by Independent Samples T-Test.

	T	*p*	Cohen’s d
R2b	−3.34	0.002	−1.01

T—Student’s *t*-test value; *p*—*p*-value—level of statistical significance (set at 0.05); Cohen’s d—effect size ratio.

**Table 3:** Results of ANOVA analysis of significance of Within Subjects Effects differences in SD of COP displacements.

	F	*p*	η²_p_
task	5.77	0.021	0.12
task × GROUP	2.47	0.124	0.06
GROUP	2.59	0.115	0.06

**Table 4:** Descriptive statistics of the studied COP and coherence parameters in both study groups.

Group	Mean ± SD	
	Old	Young
R2b	60.50 ± 17.70	81.90 ± 24.20
SD qs	4.41 ± 2.88	4.09 ± 1.25
SD lean	4.19 ± 1.36	5.40 ± 1.67
SOL/R-TIB/R 0–5 Hz qs	0.53 ± 0.11	0.52 ± 0.11
SOL/R-TIB/R 0–5 Hz lean	0.48 ± 0.08	0.49 ± 0.12
SOL/L -TIB/L 0–5 Hz qs	0.52 ± 0.10	0.54 ± 0.15
SOL/L-TIB/L 0–5 Hz lean	0.47 ± 0.09	0.50 ± 0.12
SOL/R-TIB/R 15–35 Hz qs	0.30 ± 0.07	0.35 ± 0.07
SOL/R-TIB/R 15–35 Hz lean	0.33 ± 0.04	0.36 ± 0.05
SOL/L-TIB/L 15–35 Hz qs	0.30 ± 0.03	0.36 ± 0.08
SOL/L-TIB/L 15–35 Hz lean	0.33 ± 0.03	0.37 ± 0.08

## Data Availability

Data available on request due to restrictions privacy and ethical. The data presented in this study are available on request from the corresponding author. The data are not publicly available due to privacy and ethical restrictions.

## Abbreviations

sEMG	surface electromyography
R2b	the distance between maximal and minimal position of central of pressure anterior/posterior in the 2nd phase
COP A/P	central of pressure anterior/posteriori
SD	standard deviation
LoS	limits of stability
EEG	electroencephalography
TA	tibialis anterior muscle
SOL	soleus muscle
SENIAM	Surface ElectroMyoGraphy for the Non-Invasive Assessment of Muscles
